# Tensile Behavior and Evolution of the Phases in the Al_10_Co_25_Cr_8_Fe_15_Ni_36_Ti_6_ Compositionally Complex/High Entropy Alloy

**DOI:** 10.3390/e20090646

**Published:** 2018-08-29

**Authors:** Anna Maria Manzoni, Sebastian Haas, Haneen Daoud, Uwe Glatzel, Christiane Förster, Nelia Wanderka

**Affiliations:** 1 Helmholtz-Zentrum Berlin für Materialien und Energie GmbH, Hahn-Meitner-Platz 1, D-14109 Berlin, Germany; 2 Metals and Alloys, University Bayreuth, Ludwig-Thoma-Str. 36b, D-95447 Bayreuth, Germany; 3 Neue Materialien Bayreuth GmbH, Gottlieb-Keim-Str. 60, D-95448 Bayreuth, Germany

**Keywords:** high entropy alloys, microstructure, transmission electron microscopy, compositionally complex alloys

## Abstract

Compositionally complex alloys, or high entropy alloys, are good candidates for applications at higher temperatures in gas turbines. After their introduction, the equiatomic Al_17_Co_17_Cr_17_Cu_17_Fe_17_Ni_17_ (at.%) served as a starting material and a long optimization road finally led to the recently optimized Al_10_Co_25_Cr_8_Fe_15_Ni_36_Ti_6_ (at.%) alloy, which shows promising mechanical properties. Investigations of the as-cast state and after different heat treatments focus on the evolution of the microstructure and provide an overview of some mechanical properties. The dendritic solidification provides two phases in the dendritic cores and two different ones in the interdendritic regions. Three of the four phases remain after heat treatments. Homogenization and subsequent annealing produce a γ-γ’ based microstructure, similar to Ni-based superalloys. The γ phase is Co-Cr-Fe rich and the γ’ phase is Al-Ni-Ti rich. The understanding of the mechanical behavior of the investigated alloy is supported and enhanced by the study of the different phases and their nanohardness measurements. The observations are compared with mechanical and microstructural data from commercial Ni-based superalloys, Co-based alloys, and Co-Ni-based alloys at the desired application temperature of ~800 °C.

## 1. Introduction

High entropy alloys (single phase) and compositionally complex (more phases, based on the high entropy effect) alloys have shown promising mechanical properties since the beginning of their discovery. The current trend in the development of the new high entropy alloys (HEA) or compositionally complex alloys (CCA) is focused on their use in industrial applications as a replacement for Ni-based or Co-based alloys. The latter commonly find their applications in aircraft turbines. However, there is a high demand for increasingly efficient materials, not only for aircraft turbines, but also for the next generation of USC (ultra-supercritical) power plants. In this case, the efficiency must be increased, which means that the materials need to withstand temperatures of up to 750 °C [1]. Usually, Ni- and Co-based alloys are considered good candidates. In the temperature window of 600–800 °C, the commercially used alloys are restricted to a small group, especially alloy 800, Inconel 706, and Inconel 617, which are used as turbine disks.

In order to mirror the good performance of these alloys, several optimization steps have been tested in high entropy alloys and compositionally complex alloys. To make a new alloy compete with Co- and Ni-based superalloys, its tensile properties are of high importance, but unfortunately, until now, there have only been a few works on the tensile properties of HEA and CCA [2]. For the aforementioned application as turbine disks, thermomechanical strengthening mechanisms, such as cold-rolling, which can show good results [3], are not applicable. This is also the case for several potential strengthening phases, such as oxides [4] or carbides [5], which are usually obtained by powder metallurgy. Both these phases and the non-equilibrium method have been excluded because their performance in the present type of application is inferior to a coherent strengthening phase like γ’ in γ in a cast alloy that has been carefully homogenized, as has been shown in a recent review paper [6]. This is also the strengthening mechanism that is dominant in the Ni- and Co-based superalloys.

One aspect of particular importance is the amount of γ’ phase in the γ matrix [7]. It is responsible for a high ultimate tensile strength. An increase of the γ’ volume fraction is therefore a most interesting path for material optimization, not only in superalloys, but also in compositionally complex alloys.

In 2011, we started a methodical search for the improvement of the equiatomic Al_17_Co_17_Cr_17_Cu_17_Fe_17_Ni_17_ compositionally complex alloy’s mechanical properties [8,9]. This equiatomic alloy displays at least five phases and is very brittle [8,9,10,11]. First ideas, like the reduction of the amount of Cu [12,13,14,15], the increase of Al and Cr for better oxidation properties [14,16], or the reduction of the number of phases [13,14], had to be omitted because the associated mechanical properties did not meet the expectations. Especially the idea of a single phase, which makes the concept of high entropy alloys unique [17], was disappointing because of the material’s low strength [14,15].

A change of path then had to be made, and it was decided to promote the formation of a coherent strengthening phase, i.e., the aformentioned γ-γ’ morphology. It could be obtained in the Al_8_Co_17_Cr_17_Cu_8_Fe_17_Ni_33_ alloy, after the optimum heat treatment, but the γ’ precipitates were very small (about 10 nm sized) and had a low volume fraction (about 20%); thus the alloy was extremely weak [12,14,15]. The addition of small amounts of W, Mo, and Ti in the Al_8_Co_17_Cr_14_Cu_8_Fe_17_Ni_34.8_Mo_0.1_Ti_0.1_W_1_ alloy allowed a slight increase in size of the γ’ precipitates (up to 50 nm) at a lower volume fraction (about 7%), but it had a similar weakness [15]. The search had to go on.

The systematic change in the composition of the alloy and the calculation of the equilibrium diagrams using the Thermo-Calc software (Thermo-Calc Software AB, Solna, Sweden) [18] and the TTNi7 database [19] finally led to the alloy Al_10_Co_25_Cr_8_Fe_15_Ni_36_Ti_6_, which has a wide temperature range of existence of a γ-γ’ two phase region and a high proportion of the ordered γ’ phase. The mechanical properties of this alloy are based on a stable microstructure in the temperature range up to 800 °C, as reported recently [16]. Temperature tensile strength and elongation to failure up to 800 °C [16] are even superior to those of Alloy 800 and Inconel 617 [20]. At this temperature, Al_10_Co_25_Cr_8_Fe_15_Ni_36_Ti_6_ also has better mechanical properties than other high entropy alloys and compositionally complex alloys like solid solution Co_20_Cr_20_Fe_20_Mn_20_Ni_20_ (~100 MPa, [21]) or equiatomic Al_17_Co_17_Cr_17_Cu_17_Fe_17_Ni_17_ (~180 MPa, [22]).

In order to further optimize the alloy, a wider knowledge of the microstructure and its correlation with the mechanical properties is necessary. A major point is also the reproducibility of the tensile tests, which can be explained by microstructural features. Therefore, this study focuses, on the one hand, on the microstructural characterization of the as-cast and the heat treated Al_10_Co_25_Cr_8_Fe_15_Ni_36_Ti_6_ alloy, as well as on the evolution of its phases, and on the other hand, on tensile results at room temperature and at 800 °C, and the correlation between the two parts. A combination of complementary imaging techniques at several scale levels has been employed for the characterization of the phases in the alloy (optical microscopy, scanning electron microscopy (SEM), transmission electron microscopy (TEM)). Tensile tests have been performed at room temperature and 800 °C to investigate the mechanical properties concerning strength and ductility. Nanohardness measurements of each phase have been carried out to support the mechanical properties.

## 2. Materials and Methods

### 2.1. Alloy Preparation

The Al_10_Co_25_Cr_8_Fe_15_Ni_36_Ti_6_ compositionally complex alloy (CCA) was prepared from the constituent elements of 99.99% purity. It was melted in a vacuum induction furnace and solidified in a circular ceramic mould of a 140 mm length and a 10 mm diameter. Samples were solidified directionally with a preferred [001] direction using the Bridgman method. Homogenization was carried out at 1220 °C for 20 h with a subsequent cooling in the furnace. The homogenized samples were aged at 900 °C or at 950 °C for different times, i.e., 5 and 50 h (900 °C) and 50, 100, 200, 500, and 100 h (950 °C), and then cooled down in the furnace to room temperature to prevent cracking at the grain boundaries.

### 2.2. Microstructural Observations

Specimens for optical microscopy (OM), scanning electron microscopy (SEM), X-ray diffraction (XRD), and nanohardness measurements were mechanically polished down in a final polishing step with a 50 nm sized OP-U colloidal silica suspension. Specimens for SEM investigation were etched with a solution of 3 g Mo-acid in 100 mL H_2_O, 100 mL HCl, and 100 mL HNO_3_.

SEM investigations were carried out in two SEMS: the first is a Zeiss Leo LEO Gemini 1530, equipped with an EDS spectrometer (Noran System Six; Thermo Fisher Scientific Inc., Waltham, MA, USA); and the second SEM is a Zeiss 1540EsB Cross Beam. All images were recorded at 5 keV and at working distances between 7.5 and 8.7 mm.

XRD observations were carried out in a Bruker AXS (Bruker AXS GmbH, Karlsruhe, Germany), D8 diffractometer, using Cu Kα radiation in the θ–2θ configuration.

Specimens for TEM were electropolished with a solution of 83% ethanol, 10% perchloric acid, and 7% glycerin at a temperature of −7 °C and a voltage of 30 V. A TEM Philips CM30 (ThermoFischer Scientific, Waltham, MA, USA), operated at 300 kV and equipped with an energy-dispersive X-ray spectroscopy (EDS) detector (EDAX), was used in this study. The beam size for EDS measurements was 10 nm. 

Phase volume fractions and precipitate sizes were determined in 2D using the training and classification of the Weka segmentation method [23] in the open source software Fiji [24] based on ImageJ [25,26]. In some cases, these measurements were accompanied by thresholds set on EDS mappings or OM, SEM, and TEM images taken along the [100] zone axis of the γ’-γ’ morphology were used for the most varied view on particles and the reduction of systematic errors. An average of 1000 particles per state have been analyzed, except for the as-cast and the homogenized state, where the Weka method could not be applied due to the small size. An average of 100 particles have been observed here.

### 2.3. Mechanical Tests

Vickers microhardness measurements were carried out using a Polyvar met (Reichert-Jung/Leica Microsystems, Wetzlar, Germany) microscope and a MHT-10 (Anton Paar, Graz, Austria) microhardness tester under a load of ~0.5 N for 15 s. The average microhardness value was calculated from the minimum ten indentations. Nanohardness measurements were performed in a picodentor HM500 (Helmut Fischer GmbH, Sindelfingen, Germany) under a load of 25 mN for 20 s.

Flat specimens for tensile tests were manufactured by electrical discharge machining (EDM) and the recast layer was removed by fine grinding. The square cross section of the samples was 1 × 1.9 mm^2^ and the gauge length was 8 mm. The samples were attached form-closed to ceramic Al_2_O_3_-clampings. For tests at elevated temperatures, a radiant heated, fire-proof furnace was used and the tensile tests were performed with a deformation rate of 0.01 mm/s. The strain data was recorded optically by a high-resolution camera and the force for deformation by a load cell. About four stress-strain value couples were logged in one second. Young’s modulus was determined from the stress-strain curves by determining the slope of the elastic region. 

## 3. Results and Discussion

### 3.1. Morphological Evolution

In a previous study [20], it has been found that the as-cast alloy solidifies dendritically, as is the case for most high entropy and compositionally complex alloys. After homogenization at 1220 °C for 20 h, the alloy does not show any dendritic structure. 

**Figure 1:**
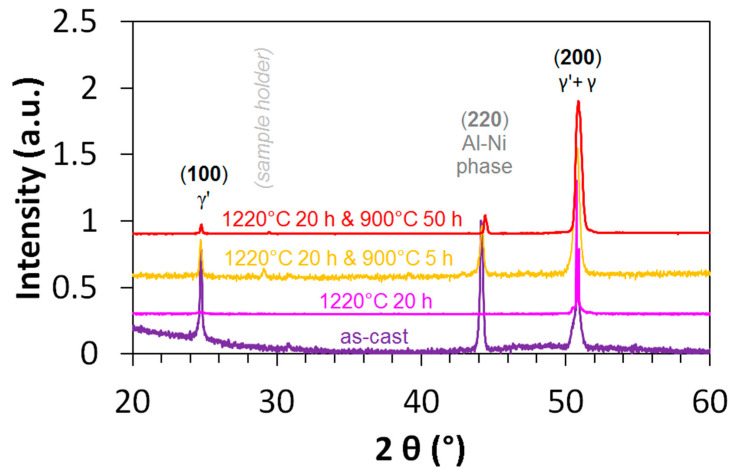
Comparison of the XRD spectra of four states of the Al_10_Co_25_Cr_8_Fe_15_Ni_36_Ti_6_ alloy.

In the same study, it has also been found that there are two main morphological features in the microstructure of the different states, i.e., coarse Al-Ni rich needles (in the annealed states) and a fine γ’ morphology (in all states). An overview of these three phases in the four states described in [20] (as-cast, homogenized, annealed 900 °C 5 h and annealed 900 °C 50 h) is given in Figure 1. The directional solidification in the [100] direction of the samples implies that the XRD spectrum records only the (100) type planes of the γ and γ’ phase and the (220) type planes of the Al-Ni rich needles, oriented 45° towards the matrix. The latter show the strongest peak in the as-cast state, because of their high volume fraction of 10 ± 3%. The Al-Ni rich phase is absent in the homogenized state (1220 °C 20 h), and in the annealed states, its peak is visible again. 

In the following, the three phases will be presented separately.

#### 3.1.1. Al-Ni Rich Needles

**Figure 2:**
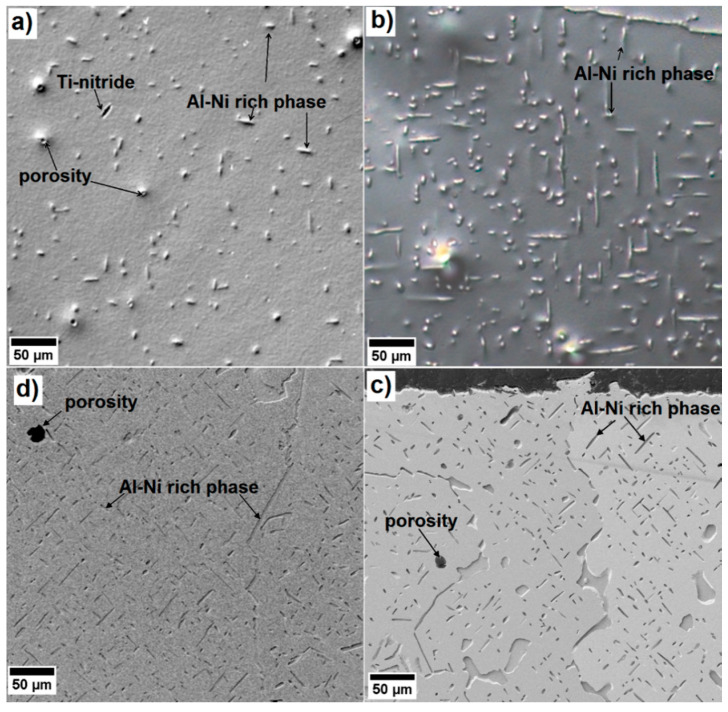
(**a**,**b**) Optical micrographs of the microstructure of the Al_10_Co_25_Cr_8_Fe_15_Ni_36_Ti_6_ alloy after annealing at (**a**) 900 °C 5 h and (**b**) 900 °C 50 h; (**c**,**d**) SEM images after annealing at (**c**) 950 °C 100 h and (**d**) 950 °C 1000 h.

Figure 2 shows the optical micrographs (a,b) and SEM images (c,d) of four exemplary annealed states of the Al_10_Co_25_Cr_8_Fe_15_Ni_36_Ti_6_ alloy. The annealing treatments at 900 °C or 950 °C lead to the formation of Al-Ni rich needle-like precipitates, displayed in bright grey in OM and dark grey in the SEM. These can reach up to 15 μm in length after 900 °C 5 h annealing (Figure 2a), and up to 40 μm after 900 °C 50 h (Figure 2b), 950 °C 100 h (Figure 2c), and 950 °C 1000 h (Figure 2d). The needles can grow into plates after the heat treatments at 950 °C, especially at the grain boundaries (Figure 2d,e). In all states of the investigated alloy, some unwanted pores and Ti-nitrides can be identified. 

These needles were not predicted by the calculation of the phase diagram in Reference [20]. They are non-existent in the homogenized state of the alloy, i.e., at 1220 °C (see [20]). They form at the lower temperatures of 900 and 950 °C. 

**Figure 3:**
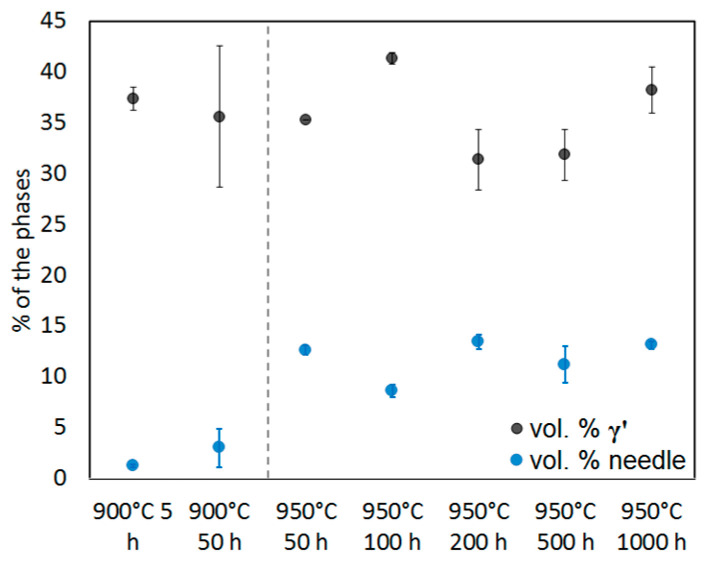
Volume fractions of the γ’ and the needle phase in the different annealing states of the Al_10_Co_25_Cr_8_Fe_15_Ni_36_Ti_6_ alloy.

The needles are oriented perpendicularly to each other. They are scarce (about 1–3 vol.%) in the 900 °C 5 h annealed alloy, but their volume fraction increases after the longer heat treatments and at a higher temperature. A comparison of the different volume fractions is shown in Figure 3. It is interesting to note that the annealing treatments at 950 °C increase the volume fraction to about 9 (100 h) or even 13% (200–1000 h). The volume fractions of needles in all states are shown in Figure 3. It can be assumed that an equilibrium is reached after 200 h at 950 °C because the volume fraction does not change after this time.

The needles’ lengths remain about the same, even after the longer heat treatments at higher temperatures of 950 °C 100 h and 950 °C 1000 h, but in some cases, the needles become plate-like, especially at the grain boundaries (see Figure 2c,d). 

#### 3.1.2. The γ-γ’ Morphology in Detail

Higher resolution observations show the second most important morphological feature in the Al_10_Co_25_Cr_8_Fe_15_Ni_36_Ti_6_ alloy, i.e., the already mentioned γ-γ’ morphology. As has been stated before, the microstructure of the samples aged at 900 °C and at 950 °C demonstrates that the two-phase microstructure (consisting of the γ and γ’ phases) obtained in the homogenized alloy and predicted by Thermo-Calc is unstable and decomposes to form the already described additional needle-like phase.

**Figure 4:**
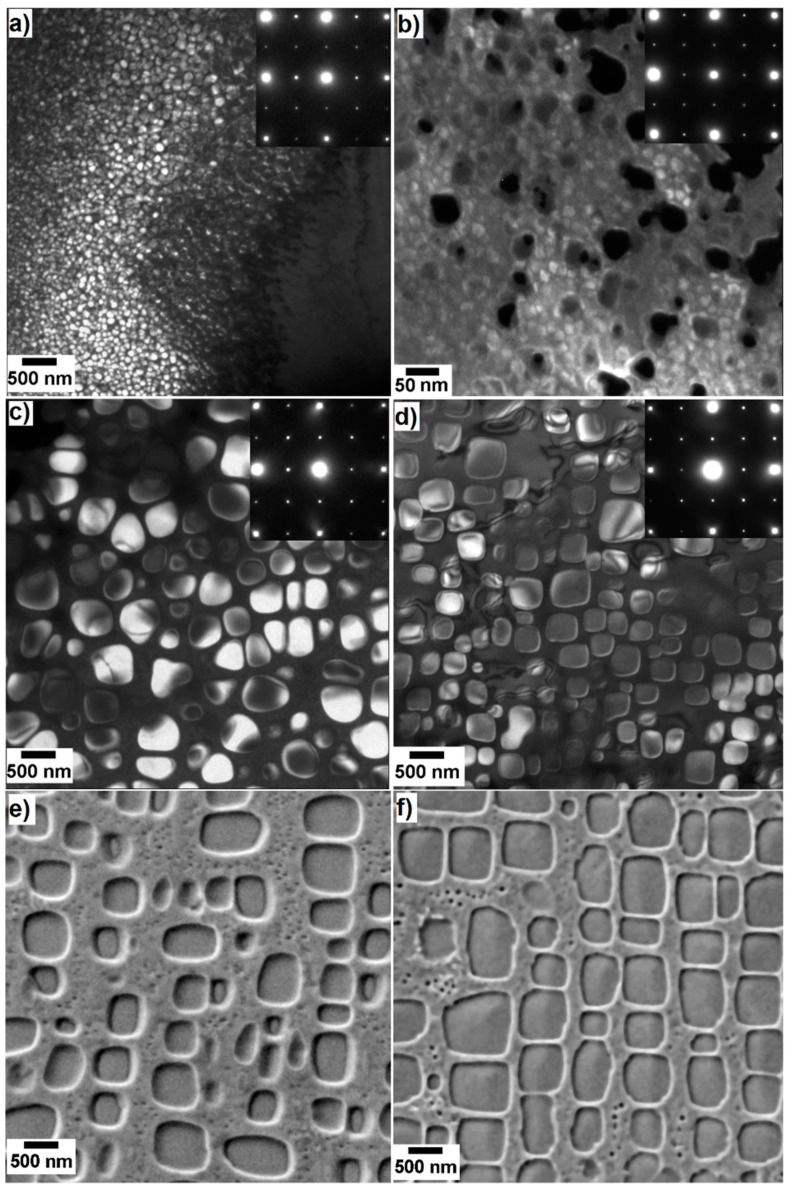
Evolution of the γ-γ’ morphology in six states of the Al_10_Co_25_Cr_8_Fe_15_Ni_36_Ti_6_ alloy: (**a**–**d**) DF TEM in (**a**) the as-cast; (**b**) homogenized at 1220 °C 20 h; subsequent annealing (**c**) at 900 °C 5 h and (**d**) at 900 °C 50 h. The upper right corner shows the corresponding SAED taken along the [001] zone axis. The DF images have been recorded with the (110) reflex; (**e**,**f**) SEM micrographs of the alloy after annealing at (**e**) 950 °C 200 h and (**f**) 950 °C 1000 h. Note the different scale bar in (**b**).

Figure 4a–d shows dark field (DF) TEM micrographs of four states of the Al_10_Co_25_Cr_8_Fe_15_Ni_36_Ti_6_ alloy, imaged using the (110) superlattice reflection. The two-phase microstructure with a γ-γ’ morphology is known from Ni-based superalloys. The brightly displayed particles are γ’ precipitates of an L1_2_ structure embedded in a darkly displayed γ matrix of a disordered A1 structure. The corresponding diffraction pattern of the [001] zone axis is shown in the inset of every micrograph. Figure 4e,f shows two SEM micrographs of the γ-γ’ morphology in the etched specimens annealed at 950 °C. These show an additional feature, i.e., small secondary γ’ particles inside the γ matrix, made visible by the etching process. They will be treated in detail in a subsequent article and will not be mentioned here again.

The primary γ’ particles have different shapes and sizes in the different states. They are rather round and 40–90 nm in diameter in the as-cast state (Figure 4a). They have undefined shapes with diameters of 6–24 nm after homogenization at 1220 °C 20 h (Figure 4b). They grow intensely after the subsequent annealing and start to adopt a cubic shape with diameters of about 200–550 nm after 900 °C 5 h (Figure 4c). They display a clear cubic shape and a diameter of 200–550 nm after 50 h annealing (Figure 4d). The heat treatments at higher temperatures and conducted for longer times further increase the particles’ sizes from 400–700 nm in the 950 °C 100 h to 400–800 nm in the 950 °C 1000 h annealed alloy.

The volume fractions of the γ’ particles in all states have been summarized in Table 1 and displayed in Figure 3. They are between 30 and 55 vol.% at all states and no tendency can be determined.

In superalloys, the size and morphology of the γ’ precipitates determine the mechanical properties. It is supposed that the same correlation can be observed in the Al_10_Co_25_Cr_8_Fe_15_Ni_36_Ti_6_ alloy.

**Figure 5:**
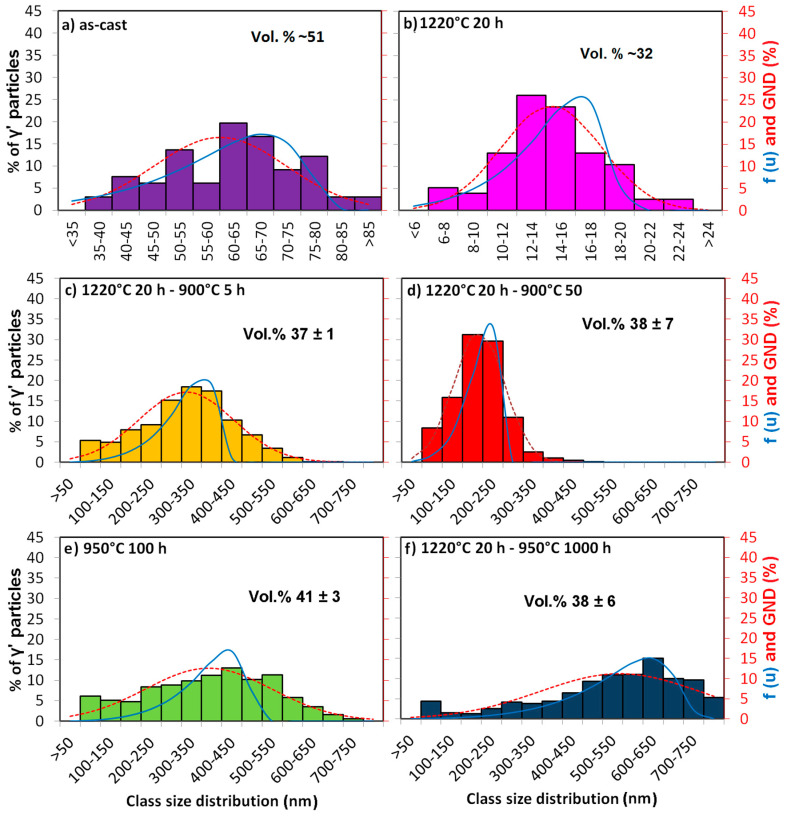
Relative γ’ particle size distribution of the Al_10_Co_25_Cr_8_Fe_15_Ni_36_Ti_6_ alloy in (**a**) the as-cast state; (**b**) homogenized state at 1220 °C 20 h; and subsequent annealing (**c**) at 900 °C 5 h, (**d**) at 900 °C 50 h, (**e**) at 950 °C 100 h, and (**f**) at 950 °C 1000 h. The dashed line corresponds to the Gauss normal distribution (GND) and the continuous line to the LSW distribution for every case. Note the different class sizes and class size distributions in the as-cast and the homogenized states. Volume fraction shave been added in the graphs for comparison.

In order to find out the growth mechanism of the γ’ precipitates, especially after ageing, we compare the two-dimensional γ’ particle size distribution for all states. The quantification of the particle size distribution (PSD) was made by characterizing about 100 (in the as-cast and homogenized states) or 1000 (in the annealed states) primary γ’ precipitates using the following method: the area was enclosed using the Weka segmentation method and, in rare cases, additionally by hand, and the area equivalent diameter *r* = √(area drawn) was obtained. The obtained values for *r* were then grouped into classes of 5 (as-cast), 2 (homogenized), or 50 nm (annealed). Their relative distribution in six selected states is shown in detail in Figure 5. The Gauss normal distribution (GND) is shown by a dotted line. Additionally, the PSD was calculated according to the LSW (Lifshitz-Slyozov-Wagner) theory of Ostwald ripening [27,28,29], following the equation [27]:

(1) $$f(u)=\frac{4u^{2}}{9}{\left(\frac{3}{3+u}\right)}^{\frac{7}{3}}{\left(\frac{-1.5}{u-1.5}\right)}^{\frac{11}{3}}\exp\left(\frac{u}{u-1.5}\right)$$

where *u* = *r*/*r*_avg_, *r* is the diameter of the analyzed precipitate, and *r*_avg_ is the average diameter of all precipitates. The LSW distribution is shown in a red continuous line, even for the as-cast and the homogenized sample, where no ripening is expected.

The PSD in the as-cast sample (Figure 5a) spreads from about 35 to about 90 nm in diameter. After homogenization, the distribution is much narrower, i.e., from about 6 to about 24 nm in diameter (Figure 4b). For the annealed states (Figure 5c–f), the PSD is widely spread and can range from 50 to 800 nm diameter. 

For all samples, the PSD fits better with the GND than with the LSW prediction. This is in accordance with research on Ostwald ripening in Ni-based alloys [30], which shows a PSD for several alloys that is broader than the one predicted by the LSW theory. A disagreement with the LSW theory has also been observed in a model Ni-14.2 Cr-5.2 Al (at.%) superalloy [31]. According to the investigations in the study from Ref. [31], the disagreement with the LSW theory can be caused by various reasons, two of which are the most plausible: (i) a transient coarsening, and (ii) the possible non-constant composition of the γ’ precipitates as a function of ageing time until it reached the equilibrium values. The last argument is in agreement with the results in the present work, where the solute concentration of Al in the γ’ precipitates of the annealed samples decreases with time from 14.0 at.% (after 900 °C 5 h ageing) to 11.4 at.% (after 900 °C 50 h), as determined by TEM-EDS, and shown in Table 1. 

**Figure 6:**
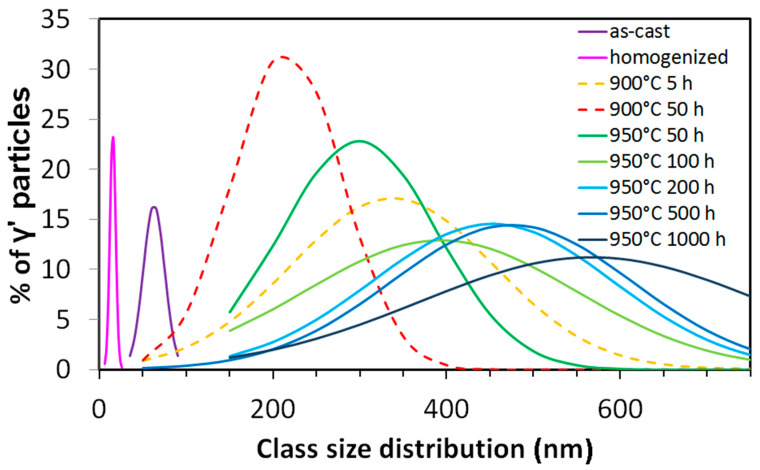
The Gauss Normal Distributions of the relative γ’ particle size distribution of the Al_10_Co_25_Cr_8_Fe_15_Ni_36_Ti_6_ alloy in all investigated states. The 900 °C annealed states are shown in dashed lines for an easier comparison.

A comparison of the GND of the γ’ sizes of all ten investigated states is shown in Figure 6. As has been shown in Figure 4a,b, the as-cast (purple) and the homogenized (pink) states stand out due to their small γ’ sizes. 

The three states after annealing at 900 °C show the most peculiar behavior. The treatment of 5 h annealing (orange dotted line) produces the largest γ’ particles, but also the most wide spread of sizes. The treatment of 50 h annealing (red dotted line) shows the smallest annealing γ’ precipitates, and the narrowest distribution. This is contradictory to the LSW theory, which predicts the growing of the precipitates’ average radius with t^1/3^. As this result is quite unexpected, several counting methods and many micrographs have been compared to a total number of over 6000 particles of these three states and the result has been confirmed by all methods. It can be concluded that the 900 °C 50 h annealing produces a unique behavior in this Al_10_Co_25_Cr_8_Fe_15_Ni_36_Ti_6_ alloy.

Unlike the annealing at 900 °C, the annealing at 950 °C produces a γ’ particle growth that is in accordance with the LSW theory of particle growth (Equation (2)):

R^3^ = R_0_^3^ + Kt (2)

where R is the average particle radius after time t, R_0_ is the initial particle radius, 3 is the exponent for diffusion-controlled growth, and K is the rate constant [32]. In our case, R_0_ corresponds to the average particle radius after homogenization, which is around 4 nm. It can be neglected compared to the radii of the particles after annealing, and thus the equation can be simplified to Equation (3):

R ∝ t^1/3^ (3)

**Figure 7:**
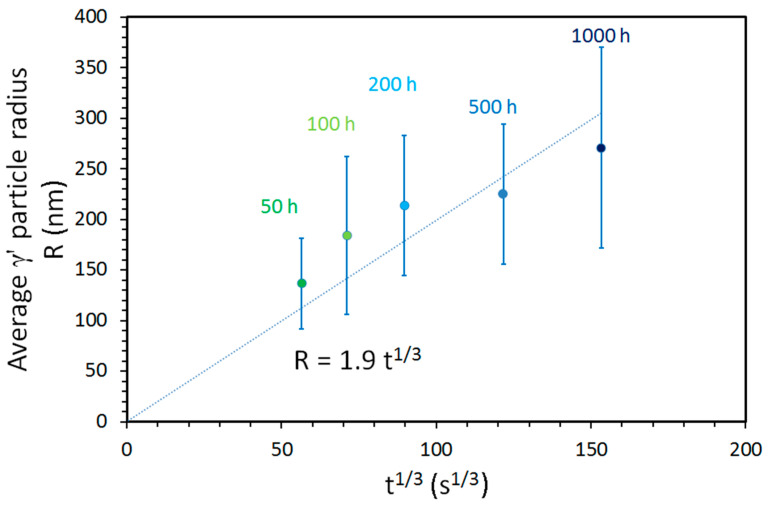
Growth of the γ’ particles in the states annealed at 950 °C, according to the LSW theory. The error bars are given by the standard deviation 2σ.

This relationship of the average γ’ particles radii versus t^1/3^ and the corresponding error bars are displayed in Figure 7. It shows the linear growth of the average particle radius with the cubic root of time, with a constant of about K = 1.9.

### 3.2. Mechanical Observations

The microstructure of the samples were correlated to tensile tests at room temperature and at 800 °C, in addition to Vickers micro- and nanohardness measurements.

#### 3.2.1. Tensile Tests

**Figure 8:**
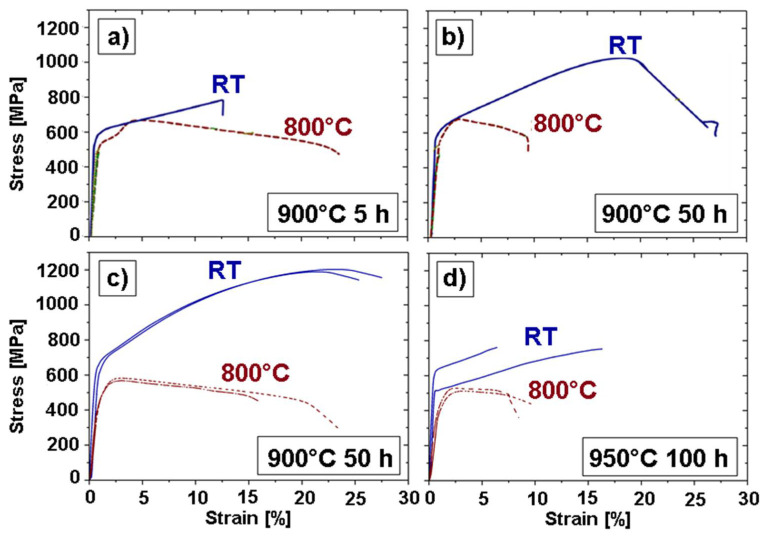
Engineering stress-strain curves of (**a**) the 900 °C 5 h (from [20]); (**b**) the 900 °C 50 h (from [20]); (**c**) the 900 °C 50 h; and (**d**) the 950 °C 100 h annealed states at room temperature and at 800 °C.

The as-cast and the homogenized state have not been submitted to tensile tests. The tensile data from the 900 °C 5 h and the 900 °C 50 h annealed states was taken from [20]. They are shown in Table 2, together with the new tensile data of the 900 °C 50 h and the 950 °C 100 h annealed states. The engineering tensile curves of the latter two are shown in Figure 8, both at room temperature and at 800 °C. 

From [20], it is known that a higher RT ultimate tensile strength σ_TS_ can be observed for the 900 °C 50 h state (1039 MPa) than for the 900 °C 5 h state (786 MPa). At a 800 °C testing temperature, their tensile strengths are almost identical (~650 MPa). 

The new experiments of the 900 °C 50 h state show slightly higher RT σ_TS_ values (~1200 MPa) and lower 800 °C σ_TS_ values (~580 MPa). 

The most interesting to note in Figure 8 is the reproducibility of the tensile stress-strain curves. It is very high in the 900 °C 50 h state, but the results for the investigated 950 °C 100 h specimens are widely spread. It is almost impossible to determine an average σ_TS_ value for the investigated specimens of the 950 °C 100 h alloy. This is even worse at RT than at 800 °C. 

**Figure 9:**
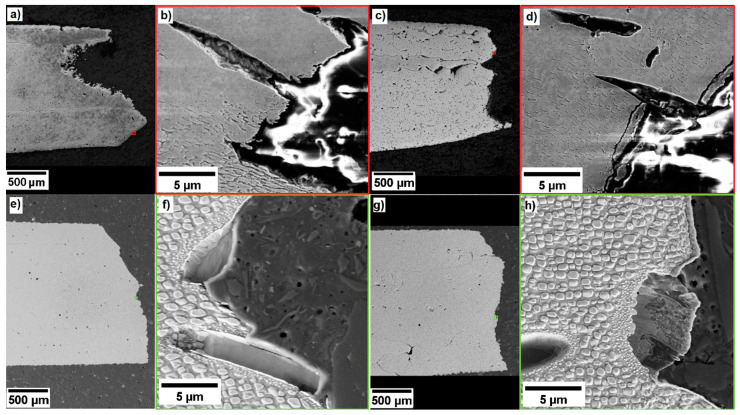
SEM images of fractures of the states (**a**–**d**) 900 °C 50 h tested at (**a**,**b**) RT and (**c**,**d**) 800 °C and (**e**–**h**) 950 °C 100 h tested at (**e**,**f**) RT and (**g**,**h**) at 800 °C. The colored boxes show the zoom to reveal the microstructure.

A reason for this difference in fracture behavior of the 900 °C and the 950 °C annealed states can be found in the microstructure of the deformed states (see Figure 9).

There are three main factors that play a role in the difference in fracture behavior:

1. The deformation temperature and the implied softening of the material
2. The abundance of the Al-Ni rich needles
3. The state of the γ’ precipitates

They will be compared in detail in the following, by their order of magnitude.

1. The deformation temperature is the most important factor. The heating from RT to 800 °C implies a softening of the material and allows for a more ductile tensile behavior. The fracture micrographs of the samples deformed at RT, shown in Figure 9a,e, show little (900 °C 50 h state, Figure 9a) or no (950 °C 100 h state, Figure 9e) necking behavior and thus a quite brittle fracture. The samples deformed at 800 °C (Figure 9c,g) display an important necking and thus a ductile fracture.

This factor is no surprise and the investigated CCA behaves like many other alloys.

2. The amount of needles is lowest in the 900 °C 5 h annealed alloy (about 1%) and highest in the 950 °C 100 h annealed alloy (about 13%). Both the longer annealing times and the higher annealing temperature increase the amount of needles, but the temperature has the stronger influence. In all five states annealed at 950 °C, the volume fraction of needles is up to ten times higher than in the 900 °C annealed states.

When observing the fracture micrographs, it was hardly possible to find any needle in the 900 °C 5 h state, and none at the fracture line (see Figure 9d). The 900 °C 50 h specimen in Figure 9b does show some needles, but the fracture seems to be uninfluenced by them because the fracture line mainly passes through the γ-γ’ region. The 950 °C states in Figure 9f,h, however, show a clear fracture preference along the needles all over the specimen. 

Note that the aforementioned secondary γ’ particles have a preference for the regions around the needles. This is not the case in the 900 °C annealed states. This observation is helpful for determining the broken needles at the fracture line—the secondary γ’ ribbons are always visible, even if the needle has quarried out.

These observations lead to the conclusion that the amount of needles is a major factor in the fracture behavior of the Al_10_Co_25_Cr_8_Fe_15_Ni_36_Ti_6_ alloy, both concerning the earlier fracture and the reproducibility of the results. Thus, in the following, the higher temperature heat treatment at 950 °C will be omitted and the next observations will be focused on the specimens annealed at the more promising heat treatments at 900 °C.

It is interesting to note that the γ’ particles in the 900 °C annealed alloys (Figure 9b,d) show a heavy deformation from their original cuboid shape. They are heavily elongated in the 900 °C 50 h annealed state after deformation at RT (Figure 9b) and they adopted a starry shape in the 900 °C 5 h annealed state after deformation at 800 °C. There is hardly any deformation of the γ’ particles visible in the 950 °C annealed states (Figure 9f,g). It can thus be concluded that the γ’ particles absorbed the plastic deformation in the 900 °C annealed alloys, while in the 950 °C annealed alloys, the important part of the deformation energy concentrated in the interface needle-matrix.

3. The γ-γ’ morphology at room temperature has been analyzed in detail (see Section 3.1.2) and is responsible for the difference in behavior between the 900 °C 5 h and the 900 °C 50 h state. As stated above, after 900 °C 5 h annealing, the γ’ precipitates have not yet established their optimum cuboidal shape. After 900 °C 50 h annealing, however, with the cuboids sides parallel to the tensile load direction and thus a higher geometrical ordering, the dislocation movement is more difficult. This effect is in accordance with the order hardening mechanism, which is often observed in Ni-based alloys [33,34].

**Figure 10:**
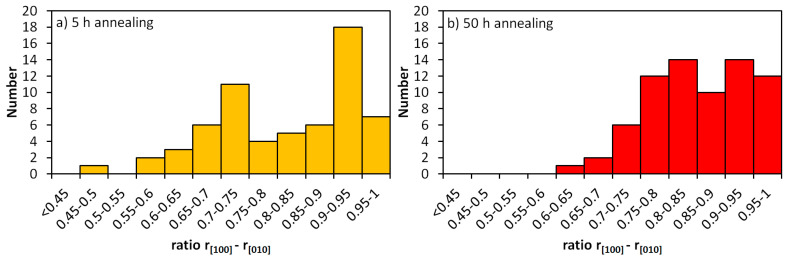
Distribution of the ratios of the γ’ precipitate lengths in the Al_10_Co_25_Cr_8_Fe_15_Ni_36_Ti_6_ alloy along the [100] and [010] directions: (**a**) after 900 °C 5 h and (**b**) after 900 °C 50 h.

To understand the morphological evolution of the γ’ precipitates between 900 °C 5 h and 50 h ageing, the aspect ratios of the γ’ precipitates were determined by using the precipitates’ diameters *r* measured in the [100] and the [010] directions. The distribution of these aspect ratios is shown in Figure 10. 

In the 900 °C 5 h annealed alloy, there is a widespread, bimodal distribution of the aspect ratios (see Figure 5c). About 37% of the analyzed precipitates have an aspect ratio below 0.75. At the same time, about 39% of them have an aspect ratio >0.9 (see Figure 10a). This result indicates that the precipitates still have no clearly defined cubic shape, as can also be seen in Figure 4c. The secondary precipitates have not been taken into account in the distribution of Figure 5c. 

The 50 h annealed alloy has a narrower distribution of aspect ratios. The maximum of the distribution shown in Figure 10b is located at about 0.85–0.9. In this case, the precipitates are close to cubic. 

The longer heat treatment allows the primary γ’ precipitates to approach their energetically most favorable shape, i.e., cuboids, which are a compromise between the lowest surface-to-volume ratio (spheres) and the lowest elastic energy (perfect cubes).

#### 3.2.2. Micro- and Nanohardness

The microhardness measurements were performed in the as-cast alloy in the dendritic region and in the annealed specimens randomly, including the Al-Ni rich needles. No microhardness change can be observed through the states as-cast homogenized and annealed at 900 °C. It remains 341–345 HV50/20 independently of the heat treatment. The microhardness is thus not much influenced by the presence of the Al-Ni rich needles nor by the average size of the γ’ precipitates, which varies from ~14 nm (homogenized) to 312 ± 55 nm (annealed at 900 °C 5 h). The volume fraction of γ’ does not contribute to the microhardness, either. The reason for the similar value of the microhardness of the samples with different states is most probably the size of the indents, which is usually about 20 µm. Thus, the measured microhardness is an average over all phases in the alloy. 

The microhardness in the state annealed at 950 °C is slightly higher (about 370 compared to about 345 HV50/20 in the other states). This can probably be linked to the significantly higher amount of Al-Ni rich needles (13 vol.% compared to 1–3 vol.%).

**Figure 11:**
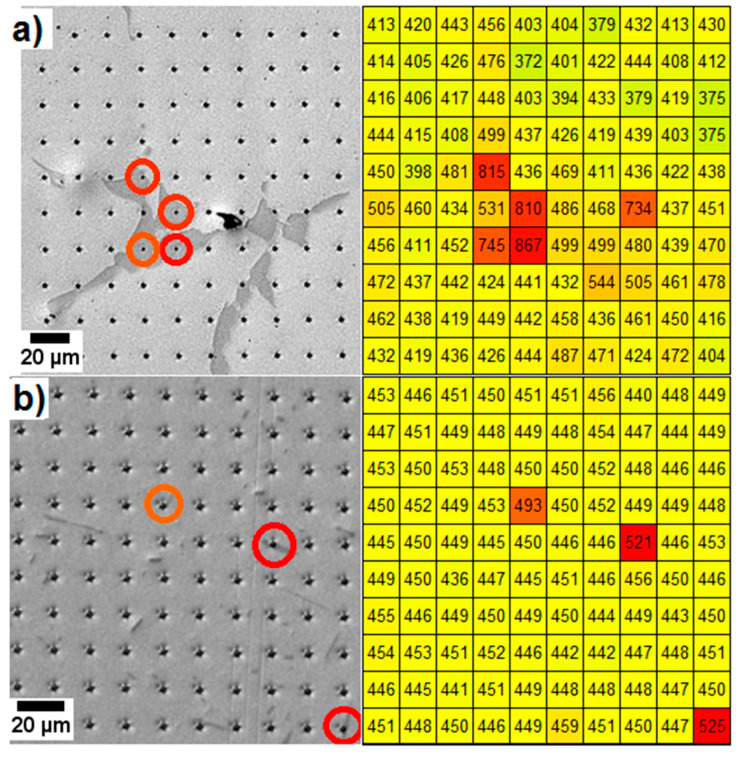
Picodentor hardness maps of two investigated states of the Al_10_Co_25_Cr_8_Fe_15_Ni_36_Ti_6_ alloy. Left column: the position of the nanoindentations; right column: the corresponding color-coded HV0.0025/20 Vickers hardness values (red = highest values). (**a**) The as-cast sample; (**b**) the sample aged at 900 °C 50 h.

A nanoindentation hardness map confirms that there are differences in hardness between the phases. Figure 11 shows a set of 100 nanoindents on the surface of two exemplary states, i.e., as-cast and annealed at 900 °C 50 h. The small load of 25 mN has been chosen in order to make sure that the influence of the small needles has the highest impact in the indent. Note that another set with a load of 100 mN shows less difference between the needles and the γ-γ’ region and is therefore not shown here. 

The as-cast alloy (Figure 11a) shows the most important local hardness changes, due to the dendritic morphology. The interdendritic regions are much harder (>700 HV0.025/20, shown in red) than the dendritic regions (~430 HV0.025/20). In the 900 °C 50 h specimen (Figure 11b), it was possible to place the indents on some Al-Ni rich needle phases (shown in red). These are harder (>500 HV0.025/20) than the γ-γ’ region (~450 HV0.025/20) around them. In order to obtain better statistics, several hardness indents were placed on various Al-Ni needles (images not shown here). The 1220 °C 20 h homogenized sample (not shown here) has a very homogeneous nanohardness of 457 ± 5 HV0.025/20. The nanohardness values of the samples aged at 900 °C 5 h and 950 °C 100 h are comparable to that of the sample aged at 900 °C 50 h. All hardness values are summarized in Table 2.

Unlike in the tensile tests, the preferred cuboidal shape of γ’ in the 900 °C 50 h annealed alloy does not have any effect. However, the dislocation movement created by an indentation is much more arbitrary than in a directional tensile test. The orientation of the dislocation lines could thus play a role in the alloy’s mechanical properties, emphasizing its anisotropic behavior.

Table 2 summarizes the γ’ precipitates’ size and the γ’ precipitates’ volume fraction, the micro- and nanohardness, the ultimate tensile strength σ_TS_ at room temperature, and the ultimate tensile strength at 800 °C for the five mechanically investigated states.

### 3.3. Comparison with Other Alloys

The microscopic data obtained in this work allows an overview of different properties for the following alloys:

- High entropy alloys Al_10_Co_25_Cr_8_Fe_15_Ni_36_Ti_6_, Co_20_Cr_20_Fe_20_Mn_20_Ni_20_, Al_8_Cr_17_Co_17_Cu_8_Fe_17_Ni_33_
- Ni-based Alloy 800H and Inconel 617, Alloy 800, and Inconel 706 single crystal Ni-based CMSX-4
- Co-based Co-9Al-9W-2Ta-0.02B
- Co-Ni based Co-30Ni-10Al-5Mo-2Ta and TMW-4M3-1.

γ’ sizes and volume fractions are compared with the ultimate tensile strength at 800 °C and are summarized in Table 3, along with additional phases. 

In the category “Size of γ’ precipitates”, the Al_10_Co_25_Cr_8_Fe_15_Ni_36_Ti_6_ alloy is comparable to Co-based Co-9Al-9W-2Ta-0.02B.

The volume fraction of the γ’ precipitates of the compared alloys can be classified into three groups: the highest volume fractions of about 70% are obtained in the single crystal CMSX-4, Co-based Co-9Al-9W-2Ta-0.02B and Co-Ni based Co-30Ni-10Al-5Mo-2Ta. An average volume fraction of 25–40% is obtained in the Al_10_Co_25_Cr_8_Fe_15_Ni_36_Ti_6_ alloy and Inconel 706. All other compared alloys have very low γ’ volume fractions, of 5% or below. 

Alloys with many additional phases, like the equiatomic Al_17_Co_17_Cr_17_Cu_17_Fe_17_Ni_17_, Inconel 706, Alloy 800, and Alloy 800H, usually have a rather low ultimate tensile strength at 800 °C. The exceptions are Inconel 617 and TMW-4M3-1. Generally speaking, a high number of phases does not seem to be advisable for an ultimate tensile strength at 800 °C. 

The ultimate tensile strength at 800 °C is highest in the single Crystal CMSX-4 and in Co-Ni-based TMW-4M3-1. The Al_10_Co_25_Cr_8_Fe_15_Ni_36_Ti_6_ alloy’s ultimate tensile strength is slightly higher, but most similar to the Co-30Ni-10Al-5Mo-2Ta, Co-9Al-9W-2Ta-0.02B, and Inconel 617. 

There seems to be a correlation between the γ’ volume fraction and the ultimate tensile strength at 800 °C. The latter would probably benefit from an increase of the γ’ volume fraction in the Al_10_Co_25_Cr_8_Fe_15_Ni_36_Ti_6_ alloy. 

Of all the eleven compared alloys, the Al_10_Co_25_Cr_8_Fe_15_Ni_36_Ti_6_ alloy shows the most similarities to the single crystal CMSX-4 alloy and the Co-9Al-9W-2Ta-0.02B alloy. The size of the γ’ precipitates, the volume fraction, and the ultimate tensile strength at 800 °C lie between those of CMSX-4 and Inconel 617. Further optimization will probably continue the improvement of the Al_10_Co_25_Cr_8_Fe_15_Ni_36_Ti_6_ alloy’s properties. We particularly expect in the future to improve the ultimate tensile strength at 800 °C by increasing the volume fraction of γ’ precipitates by an adequate heat treatment or through the addition of traces of γ’ builders. 

## 4. Summary

The Al_10_Co_25_Cr_8_Fe_15_Ni_36_Ti_6_ alloy solidifies dendritically and the dendrite cores are characterized by a two-fold morphology consisting of γ’ precipitates inside a disordered fcc matrix. The dendritic structure is not present after the homogenization at 1220 °C/20 h. However, the formation of γ’ precipitates could not be prevented in the homogenized sample. 

After ageing at 900 °C or 950 °C, a third phase joins the γ-γ’ morphology, i.e., the Al-Ni rich needles. The longer annealing times and higher annealing temperatures increase the volume fraction of the needles, from ~1% after 900 °C 5 h to ~13% after 950 °C 200 h. It stabilizes after 200 h at 950 °C.

The γ-γ’ morphology can be observed in all states from the as-cast to the 1220 °C 20 h homogenized state and the subsequently annealed states at 900 °C and 950 °C. The size of the γ’ particles is smallest after homogenization and increases drastically to over 800 nm in average diameter after the longest and hottest annealing at 950 °C 1000 h. After annealing at 950 °C, the γ’ precipitates grow according to the LSW theory of Ostwald ripening. This is not the case after annealing at 900 °C. The softest annealing at 900 °C 5 h produces larger average precipitates than the annealing at 900 °C 50 h.

Tensile tests of the chosen states 900 °C 5 h, 900 °C 50 h, and 950 °C 100 h reveal that the amount of Al-Ni rich needles is the main factor responsible for an early and unpredictable fracture. The high amount in the 950 °C treated states is thus unwanted and this annealing temperature can be excluded in the future. In the 900 °C annealed samples, the longer annealing time of 50 h intensifies the cuboidal shape of the γ’ precipitates, which implies better tensile results both at RT and at 800 °C. The small amount of needles does not seem to disturb the deformation mechanism.

Microhardness values are practically identical in all investigated states. At this scale, neither the γ-γ’ morphology nor the Al-Ni rich needles influence the hardness. However, nanohardness maps show a difference in hardness between the softer γ-γ’ region and the harder Al-Ni rich needles in the annealed alloys. 

When comparing the Al_10_Co_25_Cr_8_Fe_15_Ni_36_Ti_6_ alloy to ten other alloys, i.e., Ni-based, Co-based, Co-Ni-based, and other high entropy alloys, it could be concluded that the data from the Al_10_Co_25_Cr_8_Fe_15_Ni_36_Ti_6_ alloy is amongst the best in the categories γ’ particle size, γ’ volume fraction, and ultimate tensile strength at the desired 800 °C. The properties are approaching those of single crystal CMSX-4 and Co-based Co-9Al-9W-2Ta-0.02B at much lower costs. 

## Figures and Tables

**Table 1:** Concentration of the different phases in at.% in four states of the Al_10_Co_25_Cr_8_Fe_15_Ni_36_Ti_6_ alloy, as taken with TEM/EDS. The error bar is given by the standard deviation 2σ. (De: dendrite, ID: interdendritic region). The composition of the primary γ’ precipitates in the homogenized alloy cannot be measured because of their small sizes.

Phase	Composition (at.%)					
	Al	Co	Cr	Fe	Ni	Ti
Nominal	10	25	8	15	36	6
**As-cast**						
De -γ matrix	8.8 ± 2.2	**29.5 ± 1.8**	7.7 ± 1.2	**20.0 ± 4.2**	30.7 ± 4.3	3.2 ± 1.7
De -γ’ precipitate	12.1 ± 1.4	22.3 ± 1.9	3.1 ± 1.1	7.7 ± 1.7	**45.5 ± 2.9**	9.3 ± 1.2
ID core	**20.6 ± 2.5**	23.3 ± 1.4	3.6 ± 0.9	9.1 ± 1.2	34.3 ± 2.2	9.1 ± 0.5
**Homogenized 1220 °C 20 h**						
γ Matrix	9.3 ± 1.6	26.1 ± 0.8	7.2 ± 0.5	14.8 ± 1.0	**37.1 ± 0.8**	5.5 ± 0.5
γ’ precipitate						
**Annealed 1220 °C 20 h—900 °C 5 h**						
γ matrix	8.9 ± 1.7	**28.1 ± 1.2**	**9.4 ± 0.7**	**18.8 ± 0.9**	31.1 ± 1.1	3.6 ± 0.4
Primary γ’ precipitate	14.0 ± 1.0	21.4 ± 0.8	3.6 ± 0.4	8.2 ± 0.6	**44.4 ± 1.1**	8.3 ± 0.8
Al-Ni needle	**24.7 ± 1.9**	20.8 ± 0.7	2.9 ± 0.2	9.4 ± 0.1	36.2 ± 1.1	6.0 ± 0.2
**Annealed 1220 °C 20 h—900 °C 50 h**						
γ matrix	6.9 ± 0.6	**29.5 ± 0.5**	**9.3 ± 0.4**	**20.4 ± 0.6**	30.4 ± 1.0	3.5 ± 0.4
Primary γ’ precipitate	11.4 ± 0.6	22.5 ± 0.6	3.5 ± 0.4	8.8 ± 0.7	**45.0** **± 1.5**	8.7 ± 0.5
Al-Ni needle	**24.4 ± 1.3**	21.9 ± 1.7	3.6 ± 0.2	10.7 ± 0.4	33.9 ± 0.6	5.6 ± 0.2

**Table 2:** Mechanical and morphological data for the six investigated states of the Al_10_Co_25_Cr_8_Fe_15_Ni_36_Ti_6_ alloy (as-cast, 1220 °C 20 h, 1220 °C 20 h + 900 °C 5 h, 1220 °C 20 h + 900 °C 50 h, and 1220 °C 20 h + 950 °C 100 h). The error bars correspond to the standard deviation 2σ. DE: dendritic region; ID: interdendritic region.

	PropertyState	As-Cast	Homogenized 1220 °C-20 h	Annealed 1220 °C-20 h/900 °C-5 h	Annealed 1220 °C-20 h/900 °C-50 h	Annealed 1220 °C-20 h/950 °C-100 h
**Morphological data**	Size of primary γ’ precipitates (nm)	60 ± 12	14 ± 3	368 ± 92	315 ± 62	388 ± 132
	Volume fraction of primary γ’ precipitates	~57	~32	~45	~40	~45
	Volume fraction of the (needle-like) Al-Ni phase	~5	0	~1	~4	~9
**Mechanical data**	Micro HV50/20	343 ± 22 (DE)	341 ± 12	345 ± 14	345 ± 17	373 ± 11
	Nano HV0.025/20 γ-γ’	433 ± 32 (DE)	457 ± 5	448 ± 7	451 ± 5	454 ± 91
	Nano HV0.025/20 Al-Ni phase	736 ± 87 (ID)	-	569 ± 61	560 ± 148	647 ± 72
	Ultimate tensile strength σ_TS_ at RT (MPa)	-	-	786 from [20]	1197 ± 6	758 ± 40
	Yield strength at RT (MPa)	-	-	568 from [20]	648 ± 1	473 ± 38
	Elongation at RT (%)			12 from [20]	26 ± 1	5 ± 1
	Young’s modulus at RT (GPa)	-	-	90 ± 18	99 ± 37	118 ± 32
	Ultimate tensile strength σ_TS_ at 800 °C (MPa)	-	-	672 from [20]	575 ± 7	493 ± 40
	Yield strength at 800 °C (MPa)	-	-	535 from [20]	450	310 ± 15
	Elongation to fracture at 800 °C (%)	-	-	27 from [20]	20 ± 4	14 ± 5
	Young’s modulus at 800 °C (GPa)	-	-	52 ± 6	54 ± 4	51 ± 10

**Table 3:** Comparison of different microstructural and mechanical parameters of Al_10_Co_25_Cr_8_Fe_15_Ni_36_Ti_6_ and other (commercial) alloys.

	Alloy	Size of the Primary γ’ Precipitates (after Optimum Heat Treatment)	Volume Fraction of the Primary γ’ Precipitates (after Optimum Heat Treatment)	Additional Phases (except γ Matrix)	Ultimate Tensile Strength at 800 °C (MPa)	Ref.
HEAS	Al_8_Cr_17_Co_17_Cu_8_Fe_17_Ni_33_	20 nm	20%	L1_2_ at grain boundaries	Not known	[14,15]
	Al_10_Co_25_Cr_8_Fe_15_Ni_36_Ti_6_	191 nm	36%	Al-Ni rich needles	565	this work
	Co_20_Cr_20_Fe_20_Mn_20_Ni_20_	-	-	-	~170	[21]
	Al_17_Co_17_ Cr_17_Cu_17_Fe_17_Ni_17_	-	-	A2, B2, fcc1, fcc2, L1_2_	180	[22]
Ni-based alloys	CMSX-4	500 nm	70%	-	~1100	[33,35]
	Inconel 617	80 nm	5%	Ni_2_(Cr,Mo); MC; M_23_C_6_; M_6_C	~500	[36,37]
	Inconel 706	10–100 nm	<25%	γ″, η, carbides, nitrides, (Ni_3_Nb)	Not known	[38]
	Alloy 800	<10 nm	1%	Cr_23_C_6_, TiC	~180	[39,40,41]
	Alloy 800H	<100 nm	small	Cr_23_C_6_, TiC	~200	[39,42]
Co-based alloy	Co-9Al-9W-2Ta-0.02B	~200 nm	~70%	-	~580	[43,44]
Co-Ni based alloys	Co-30Ni-10-A-5Mo-2Ta	30–50 nm	~74%	-	>600	[45]
	TMW-4M3-1	<2.5 μm	17%	Secondary & tertiary γ’, σ, η	below 1122	[46,47]

## References

[1] Di Martino S.F., Faulkner R.G., Hogg S.C., Vujic S., Tassa O. (2014). Characterisation of microstructure and creep properties of alloy 617 for high-temperature applications. Mater. Sci. Eng. A Struct. Mater. Prop. Microstruct. Process..

[2] Zhang W.R., Liaw P.K., Zhang Y. (2018). Science and technology in high-entropy alloys. Sci. China Mater..

[3] Klimova M., Stepanov N., Shaysultanov D., Chernichenko R., Yurchenko N., Sanin V., Zherebtsov S. (2018). Microstructure and mechanical properties evolution of the al, c-containing cocrfenimn-type high-entropy alloy during cold rolling. Materials.

[4] Liu X.Y., Yin H., Xu Y. (2017). Microstructure, Mechanical and tribological properties of oxide dispersion strengthened high-entropy alloys. Materials.

[5] Nam S., Kim M.J., Hwang J.Y., Choi H. (2018). Strengthening of Al0.15CoCrCuFeNiTix–C (x = 0, 1, 2) high-entropy alloys by grain refinement and using nanoscale carbides via powder metallurgical route. J. Alloys Compd..

[6] Manzoni A.M., Glatzel U. (2018). New multiphase compositionally complex alloys driven by the high entropy alloy approach. Mater. Charact..

[7] Durand-Charre M. (1997). The Microstructure of Superalloys.

[8] Singh S., Wanderka N., Kiefer K., Siemensmeyer K., Banhart J. (2011). Effect of decomposition of the Cr–Fe–Co rich phase of AlCoCrCuFeNi high entropy alloy on magnetic properties. Ultramicroscopy.

[9] Singh S., Wanderka N., Murty B.S., Glatzel U., Banhart J. (2011). Decomposition in multi-component AlCoCrCuFeNi high-entropy alloy. Acta Mater..

[10] Tong C.J., Chen M.R., Chen S.K., Yeh J.W., Shun T.T., Lin S.J., Chang S.Y. (2005). Mechanical performance of the Al x CoCrCuFeNi high-entropy alloy system with multiprincipal elements. Metall. Mater. Trans. A.

[11] Tong C.J., Chen Y.L., Chen S.K., Yeh J.W., Shun T.T., Tsau C.H., Lin S.J., Chang S.Y. (2005). Microstructure characterization of Al x CoCrCuFeNi high-entropy alloy system with multiprincipal elements. Metall. Mater. Trans. A.

[12] Daoud H.M., Manzoni A., Völkl R., Wanderka N., Glatzel U. (2013). Microstructure and tensile behavior of Al8Co17Cr17Cu8Fe17Ni33 (at.%) high-entropy alloy. JOM.

[13] Manzoni A., Daoud H., Völkl R., Glatzel U., Wanderka N. (2013). Phase separation in equiatomic AlCoCrFeNi high-entropy alloy. Ultramicroscopy.

[14] Manzoni A., Daoud H., Mondal S., van Smaalen S., Völkl R., Glatzel U., Wanderka N. (2013). Investigation of phases in Al_23_Co_15_Cr_23_Cu_8_Fe_15_Ni_16_ and Al_8_Co_17_Cr_17_Cu_8_Fe_17_Ni_33_ high entropy alloys and comparison with equilibrium phases predicted by Thermo-Calc. J. Alloys Compd..

[15] Manzoni A.M., Daoud H.M., Voelkl R., Glatzel U., Wanderka N. (2015). Influence of W, Mo and Ti trace elements on the phase separation in Al_8_Co_17_Cr_17_Cu_8_Fe_17_Ni_33_ based high entropy alloy. Ultramicroscopy.

[16] Daoud H.M., Manzoni A.M., Völkl R., Wanderka N., Glatzel U. (2015). Oxidation Behavior of Al_8_Co_17_Cr_17_Cu_8_Fe_17_Ni_33_, Al_23_Co_15_Cr_23_Cu_8_Fe_15_Ni_15_, and Al_17_Co_17_Cr_17_Cu_17_Fe_17_Ni_17_ Compositionally Complex Alloys (High-Entropy Alloys) at Elevated Temperatures in Air. Adv. Eng. Mater..

[17] Jien-Wei Y. (2006). Recent progress in high entropy alloys. Ann. Chim. Sci. Mat..

[18] The Version Tccr.

[19] (2006). Thermotech Ni-Based Superalloys Database, TTNi7, 7.0.

[20] Daoud H.M., Manzoni A.M., Wanderka N., Glatzel U. (2015). High-temperature tensile strength of Al_10_Co_25_Cr_8_Fe_15_Ni_36_Ti_6_ compositionally complex alloy (high-entropy alloy). JOM.

[21] Otto F., Dlouhy A., Somsen C., Bei H., Eggeler G., George E.P. (2013). The influences of temperature and microstructure on the tensile properties of a CoCrFeMnNi high-entropy alloy. Acta Mater..

[22] Kuznetsov A.V., Shaysultanov D.G., Stepanov N.D., Salishchev G.A., Senkov O.N. (2012). Tensile properties of an AlCrCuNiFeCo high-entropy alloy in as-cast and wrought conditions. Mater. Sci. Eng. A Struct. Mater. Prop. Microstruct. Process..

[23] Arganda-Carreras I., Kaynig V., Rueden C., Eliceiri K.W., Schindelin J., Cardona A., Seung H.S. (2017). Trainable weka segmentation: A machine learning tool for microscopy pixel classification. Bioinformatics.

[24] Schindelin J., Arganda-Carreras I., Frise E., Kaynig V., Longair M., Pietzsch T., Preibisch S., Rueden C., Saalfeld S., Schmid B. (2012). Fiji: An open-source platform for biological-image analysis. Nat. Methods.

[25] Rueden C.T., Schindelin J., Hiner M.C., DeZonia B.E., Walter A.E., Arena E.T., Eliceiri K.W. (2017). ImageJ2: ImageJ for the next generation of scientific image data. BMC Bioinform..

[26] Schneider C.A., Rasband W.S., Eliceiri K.W. (2012). Nih image to imagej: 25 years of image analysis. Nat. Methods.

[27] Wagner C. (1961). Theorie der Alterung von Niederschlägen durch Umlösen (Ostwald-Reifung). Z. Elektrochem Ber. Bunsenges. Phys. Chem..

[28] Lifshitz I.M., Slyozov V.V. (1961). The kinetics of precipitation from supersaturated solid solutions. J. Phys. Chem. Solids.

[29] Kahlweit M. (1975). Ostwald ripening of precipitates. Adv. Colloid Interface Sci..

[30] Baldan A. (2002). Review progress in Ostwald ripening theories and their applications to nickel-base superalloys Part I: Ostwald ripening theories. J. Mater. Sci..

[31] Sudbrack C.K., Yoon K.E., Mao Z., Noebe R.D., Isheim D., Seidman D.N. (2003). Temporal Evolution of Nanostructures in a Model Nickel-Base Superalloy: Experiments and Simulations.

[32] Gleiter H., Cahn R.W., Hassen P. (1983). Microstructure. Physical Metallurgy.

[33] Sengupta A., Putatunda S.K., Bartosiewicz L., Hangas J., Nailos P.J., Peputapeck M., Alberts F.E. (1994). Tensile behavior of a new single-crystal nickel-based superalloy (CMSX-4) at room and elevated temperatures. J. Mater. Eng. Perform..

[34] Müller L., Glatzel U., Feller-Kniepmeier M. (1993). Calculation of the internal stresses and strains in the microstructure of a single crystal nickel-base superalloy during creep. Acta Metall. Mater..

[35] Reed R.C. (2006). The Superalloys. Fundamentals and Applications.

[36] Wu Q.Y., Song H.J., Swindeman R.W., Shingledecker J.P., Vasudevan V.K. (2008). Microstructure of long-term aged in617 Ni-base superalloy. Metal. Mater. Trans. A Phys. Met. Mater. Sci..

[37] Inconel Alloy 617. http://www.Specialmetals.Com/documents/inconel%20alloy%20617.Pdf.

[38] Wanderka N., Naundorf V., Banhart J., Mukherji D., Genovesse D.D., Rosler J. (2004). Microstructural characterization of Inconel 706 alloy. Surf. Interface Anal..

[39] Alloy 800. http://www.Sandmeyersteel.Com/images/alloy-800-spec-sheet.Pdf.

[40] Coppola R., Fiorentin S.R. (1987). Study of γ′-precipitation kinetics in alloy 800 at 575 °C by small angle neutron scattering. Nucl. Instr. Meth. Phys. Res. Sect. B Beam Interact. Mater. Atoms.

[41] Vittori M. (1981). Gamma particle coarsening and yield in alloy 800. J. Mater. Sci..

[42] Nilsson J.O., Thorvaldsson T. (1985). Low cycle fatigue behavior of alloy 800 h at 600–800 °C—Effect of grain size and gamma particle dispersion. Fatigue Fracture Eng. Mater. Struct..

[43] Zhong F., Li S.S., Sha J.B. (2015). Tensile behaviour of Co–Al–W–Ta–B–Mo alloys with a coherent γ/γ′ microstructure at room and high temperatures. Mater. Sci. Eng. A Struct. Mater. Prop. Microstruct. Process..

[44] Feng G., Li H., Li S.S., Sha J.B. (2012). Effect of Mo additions on microstructure and tensile behavior of a Co–Al–W–Ta–B alloy at room temperature. Scripta Mater..

[45] Makineni S.K., Samanta A., Rojhirunsakool T., Alam T., Nithin B., Singh A.K., Banerjee R., Chattopadhyay K. (2015). A new class of high strength high temperature cobalt based γ–γ′ Co–Mo–Al alloys stabilized with Ta addition. Acta. Mater..

[46] Gu Y., Zhong Z., Yuan Y., Osada T., Cui C., Yokokawa T., Harada H. An advanced cast-and-wrought superalloy (TMW-4M3) for turbine disk applications beyond 700 C. Proceedings of the International Symposium on Superalloys.

[47] Osada T., Gu Y.F., Nagashima N., Yuan Y., Yokokawa T., Harada H. (2013). Optimum microstructure combination for maximizing tensile strength in a polycrystalline superalloy with a two-phase structure. Acta Mater..

